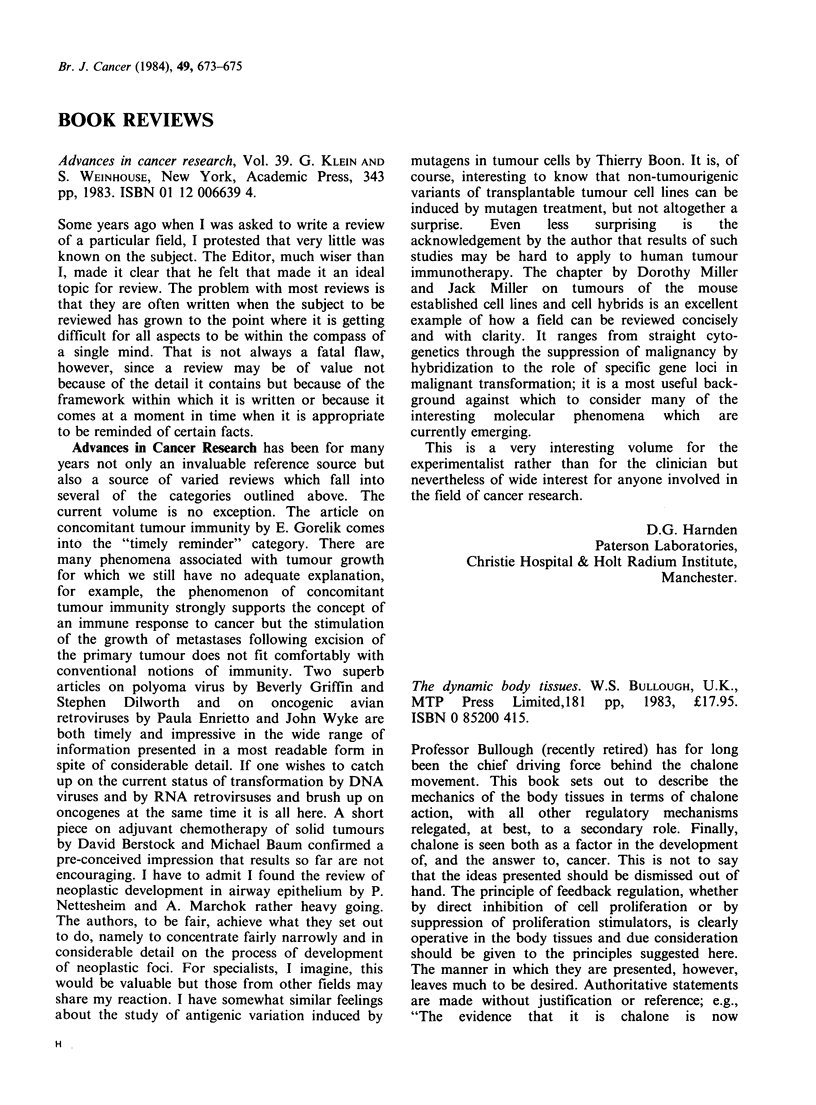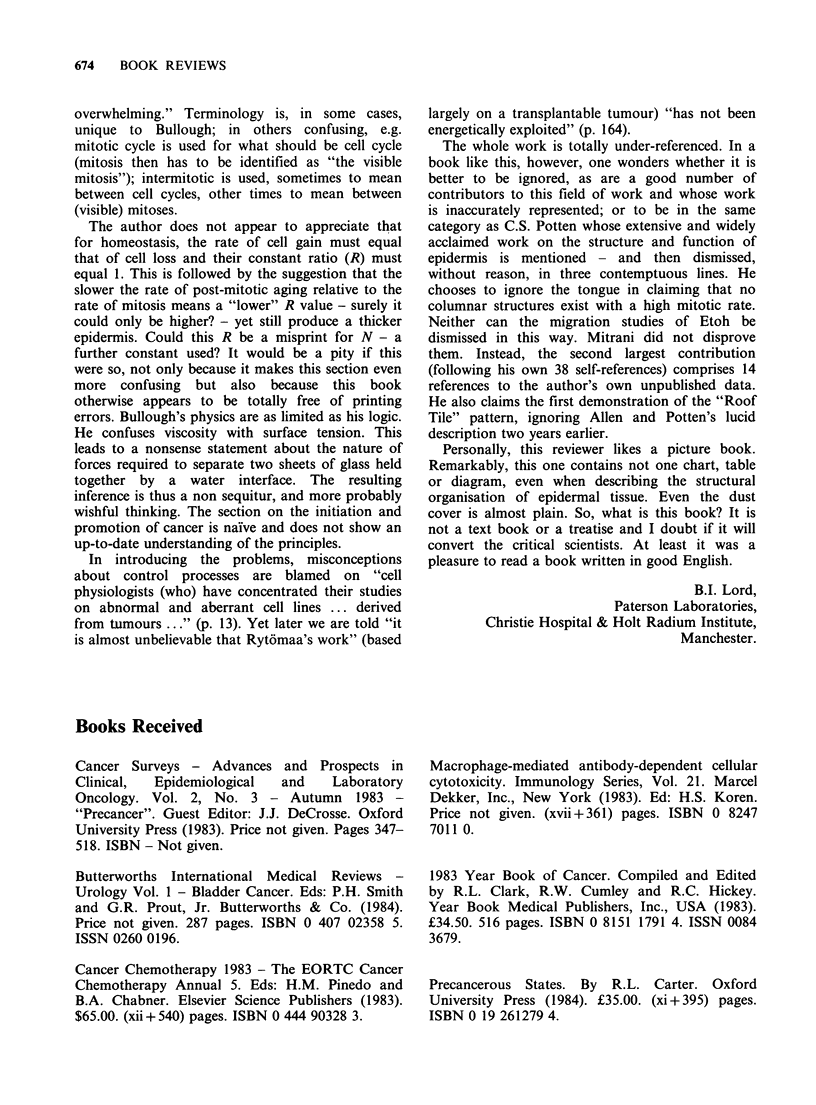# The dynamic body tissues

**Published:** 1984-05

**Authors:** B.I. Lord


*The dynamic body tissues*. W.S. Bullough, U.K., MTP Press Limited,181 pp, 1983, £17.95. ISBN 0 85200 415.

Professor Bullough (recently retired) has for long been the chief driving force behind the chalone movement. This book sets out to describe the mechanics of the body tissues in terms of chalone action, with all other regulatory mechanisms relegated, at best, to a secondary role. Finally, chalone is seen both as a factor in the development of, and the answer to, cancer. This is not to say that the ideas presented should be dismissed out of hand. The principle of feedback regulation, whether by direct inhibition of cell proliferation or by suppression of proliferation stimulators, is clearly operative in the body tissues and due consideration should be given to the principles suggested here. The manner in which they are presented, however, leaves much to be desired. Authoritative statements are made without justification or reference; e.g., "The evidence that it is chalone is now overwhelming." Terminology is, in some cases, unique to Bullough; in others confusing, e.g. mitotic cycle is used for what should be cell cycle (mitosis then has to be identified as "the visible mitosis"); intermitotic is used, sometimes to mean between cell cycles, other times to mean between (visible) mitoses.

The author does not appear to appreciate that for homeostasis, the rate of cell gain must equal that of cell loss and their constant ratio (*R*) must equal 1. This is followed by the suggestion that the slower the rate of post-mitotic aging relative to the rate of mitosis means a "lower" *R* value – surely it could only be higher? – yet still produce a thicker epidermis. Could this *R* be a misprint for *N* – a further constant used? It would be a pity if this were so, not only because it makes this section even more confusing but also because this book otherwise appears to be totally free of printing errors. Bullough's physics are as limited as his logic. He confuses viscosity with surface tension. This leads to a nonsense statement about the nature of forces required to separate two sheets of glass held together by a water interface. The resulting inference is thus a non sequitur, and more probably wishful thinking. The section on the initiation and promotion of cancer is naïve and does not show an up-to-date understanding of the principles.

In introducing the problems, misconceptions about control processes are blamed on "cell physiologists (who) have concentrated their studies on abnormal and aberrant cell lines ... derived from tumours ..." (p. 13). Yet later we are told "it is almost unbelievable that Rytömaa's work" (based largely on a transplantable tumour) "has not been energetically exploited" (p. 164).

The whole work is totally under-referenced. In a book like this, however, one wonders whether it is better to be ignored, as are a good number of contributors to this field of work and whose work is inaccurately represented; or to be in the same category as C.S. Potten whose extensive and widely acclaimed work on the structure and function of epidermis is mentioned – and then dismissed, without reason, in three contemptuous lines. He chooses to ignore the tongue in claiming that no columnar structures exist with a high mitotic rate. Neither can the migration studies of Etoh be dismissed in this way. Mitrani did not disprove them. Instead, the second largest contribution (following his own 38 self-references) comprises 14 references to the author's own unpublished data. He also claims the first demonstration of the "Roof Tile" pattern, ignoring Allen and Potten's lucid description two years earlier.

Personally, this reviewer likes a picture book. Remarkably, this one contains not one chart, table or diagram, even when describing the structural organisation of epidermal tissue. Even the dust cover is almost plain. So, what is this book? It is not a text book or a treatise and I doubt if it will convert the critical scientists. At least it was a pleasure to read a book written in good English.

B.I. Lord,
Paterson Laboratories,
Christie Hospital & Holt Radium Institute,
Manchester.